# Evidence-based universal health coverage interventions delivery in infectious disease of poverty elimination and eradication

**DOI:** 10.1186/s40249-023-01169-x

**Published:** 2024-01-02

**Authors:** Ernest Tambo, Chidiebere E. Ugwu, Amberbir Alemayehu, Anil Krishna, Agnes Binagwaho

**Affiliations:** 1 Center for Leadership in Global Health Equity, University of Global Health Equity, Kigali, Rwanda; 2 Africa Disease Intelligence, Preparedness and Response (ADIPR), Yaoundé, Cameroon; 3 Higher Institute of Health Sciences, Faculty of Medicine, Bangangte, Cameroon; 4 Institute for Global Health Equity Research, University of Global Health Equity, Kigali, Rwanda; 5 Department of Human Biochemistry, Nnamdi Azikiwe University, Nnewi, Anambra Nigeria

## Abstract

The recent 78th United Natiions General Assembly (UNGA) declaration relaunched a new health priority and political commitment in accelerating Universal Health Coverage (UHC), adopted by the United Nations (UN) in 2015 and are intended to be met by 2030. At mid-way point of UHC implementation, we advocate for the much needed programmatic implementation research, evidence-based interventions (EBIs) policies and strategies leadership and management capacity building to enable robust, resilient and sustainable multi-sectoral partnerships, integrated coordination and governance mechanisms capabilities in accelerating UHC package mainly infectious diseases of poverty elimination and eradication agenda.

**Graphical Abstract:**

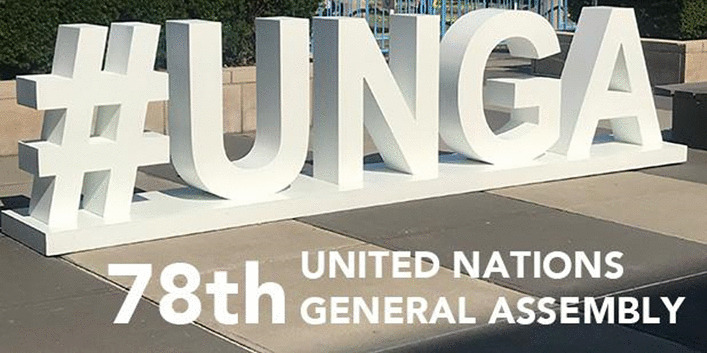

The recent 78th United Nations General Assembly (UNGA) declaration re-established a new health priority and political commitment in accelerating Universal Health Coverage (UHC), adopted by the UN in 2015 and are intended to be met by 2030 [1]. The declaration highlights the urgent need to invest in UHC-health systems resilience, and the delivery of continuum access to and uptake of full range of quality and essential healthcare services to populations needs. This should be inclusive, equitable and sustainable without having financial hardship. Moreover, it is critical to strengthen health systems resiliency, early preparedness and timely emergency response to global and local public health emergencies such as COVID-19 pandemic [1, 2].

At mid-way point of UHC implementation, we advocate for the much-needed implementation research, evidence-based interventions (EBIs) policies, strategic leadership and health systems management capacity building [3]. A robust, resilient, and sustainable multi-sectorial partnerships is urgently needed. Building a well-coordinated governance mechanisms capabilities is required in accelerating UHC package mainly in infectious diseases of poverty (malaria, tuberculosis, poliomyelitis, leprosy, rabies, leishmaniasis, and others neglected tropical diseases elimination and eradication agenda [2–4]. More so, in the global south where these diseases burden are disproportionately high and ambivalently managed this far [1, 2]. Concerted ample investment is paramount to improve the health financing mechanisms and resource allocation, and capacitating on local leadership and financing management resiliency know-how, through decentralized health system and accountability culture efforts. The capacities strengthening need cannot be overemphasized for scale and sustainable access to uptake of and satisfaction to quality and sustainable UHC packages and service delivery, and impact outcomes [2–4].

Prioritizing data-oriented culture to evidence-based policy and practice, and implementation research that produce the evidence needed in building resilient and sustainable primary healthcare system innovations premised on quality community-based UHC delivery, satisfaction and social protection policies [1, 2, 5]. Moreover, leveraging on proven efficacy and effectiveness lessons learned from EBIs, implementation research in fast-tracking infectious diseases elimination and eradication agenda, while addressing contextual challenges critical in monitoring national UHC attainment through advocacy and promotion of prevention, elimination and eradication EBIs implementation.

Investing in robust and sustainable global health security collaboration and coordinated response is core in strengthening public health surveillance and resilient health system performance and outcome at all levels, with partners’ technical assistance and implementation support. Also, bolstering on digitalization and artificial intelligence, boosting community health insurance and other new technologies opportunities in optimizing evidence-based primary healthcare programs demand and need solutions, particularly in remote and marginalized populations.

## Data Availability

All data are available and additional resources could be requested.

## Abbreviations

COVID-19: Coronavirus diseases 2019
EBIs: Evidence-based interventions
NTDs: Neglected Tropical Diseases
UNGA: United Nations General Assembly
UHC: Universal Health Coverage
